# The Quantum Chemical Cluster Approach in Biocatalysis

**DOI:** 10.1021/acs.accounts.2c00795

**Published:** 2023-03-28

**Authors:** Xiang Sheng, Fahmi Himo

**Affiliations:** † Tianjin Institute of Industrial Biotechnology, Chinese Academy of Sciences, Tianjin 300308, PR China; ‡ National Center of Technology Innovation for Synthetic Biology, Tianjin 300308, PR China; § Department of Organic Chemistry, Arrhenius Laboratory, Stockholm University, SE-10691 Stockholm, Sweden

## Abstract

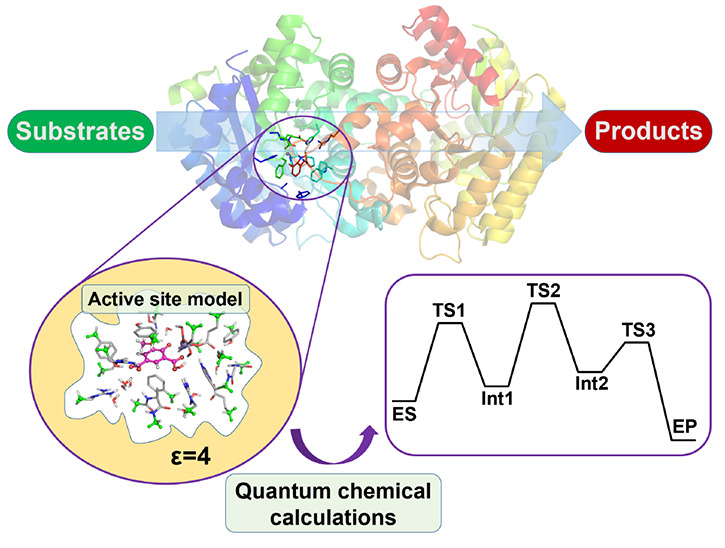

The quantum chemical cluster approach has been used for modeling enzyme active sites and reaction mechanisms for more than two decades. In this methodology, a relatively small part of the enzyme around the active site is selected as a model, and quantum chemical methods, typically density functional theory, are used to calculate energies and other properties. The surrounding enzyme is modeled using implicit solvation and atom fixing techniques. Over the years, a large number of enzyme mechanisms have been solved using this method. The models have gradually become larger as a result of the faster computers, and new kinds of questions have been addressed. In this Account, we review how the cluster approach can be utilized in the field of biocatalysis. Examples from our recent work are chosen to illustrate various aspects of the methodology. The use of the cluster model to explore substrate binding is discussed first. It is emphasized that a comprehensive search is necessary in order to identify the lowest-energy binding mode(s). It is also argued that the best binding mode might not be the productive one, and the full reactions for a number of enzyme–substrate complexes have therefore to be considered to find the lowest-energy reaction pathway. Next, examples are given of how the cluster approach can help in the elucidation of detailed reaction mechanisms of biocatalytically interesting enzymes, and how this knowledge can be exploited to develop enzymes with new functions or to understand the reasons for lack of activity toward non-natural substrates. The enzymes discussed in this context are phenolic acid decarboxylase and metal-dependent decarboxylases from the amidohydrolase superfamily. Next, the application of the cluster approach in the investigation of enzymatic enantioselectivity is discussed. The reaction of strictosidine synthase is selected as a case study, where the cluster calculations could reproduce and rationalize the selectivities of both the natural and non-natural substrates. Finally, we discuss how the cluster approach can be used to guide the rational design of enzyme variants with improved activity and selectivity. Acyl transferase from *Mycobacterium smegmatis* serves as an instructive example here, for which the calculations could pinpoint the factors controlling the reaction specificity and enantioselectivity. The cases discussed in this Account highlight thus the value of the cluster approach as a tool in biocatalysis. It complements experiments and other computational techniques in this field and provides insights that can be used to understand existing enzymes and to develop new variants with tailored properties.

## Key References

- ShengX.; HimoF.Theoretical Study of Enzyme Promiscuity: Mechanisms of Hydration and Carboxylation Activities of Phenolic Acid Decarboxylase. ACS Catal.2017, 7, 1733–1741.^1^*In this paper, the cluster approach was used to evaluate a large number of binding modes of the non-natural substrates and to work out the mechanisms of two promiscuous reactions of phenolic acid decarboxylase.*
- ShengX.; HimoF.Enzymatic Pictet–Spengler Reaction: Computational Study of the Mechanism and Enantioselectivity of Norcoclaurine Synthase. J. Am. Chem. Soc.2019, 141, 11230–112383126526810.1021/jacs.9b04591.^2^*This paper is an example of how the cluster approach can be used for studying enantioselectivity of enzymes. It highlights the importance of considering the entire reaction pathway to elucidate the origins of the selectivity.*
- KazemiM.; ShengX.; HimoF.Origins of Enantiopreference of *Mycobacterium smegmatis* Acyl Transferase: A Computational Analysis. Chem.–Eur. J.2019, 25, 11945–119543129450010.1002/chem.201902351.^3^*In this study, the cluster approach was used to provide a framework for the understanding of the enantiopreference of acyl transferase from Mycobacterium smegmatis. The insights provided by the calculations were later used to guide the rational design of enzyme variants with improved properties.*
- ShengX.; KazemiM.; Ża̧dło-DobrowolskaA.; KroutilW.; HimoF. Mechanism of Biocatalytic Friedel–Crafts Acylation by Acyltransferase from *Pseudomonas Protegens*. ACS Catal.2020, 10, 570–5773192994710.1021/acscatal.9b04208PMC6945686.^4^*In this paper, a large cluster model was used to work out the detailed mechanism of acyltransferase from Pseudomonas Protegens, where a residue located far from the substrate could be identified as the general acid/base in the reaction.*

## 1 Introduction

Computational chemistry methods are today of great value in the field of biocatalysis. A number of techniques are routinely used both to rationalize experimental findings and also to predict and help to rationally design enzymes with new reactivities and properties. These methods include, e.g., molecular docking, molecular dynamics (MD), empirical valence bond (EVB), hybrid quantum mechanics/molecular mechanics (QM/MM), and quantum chemical cluster calculations.^5−29^ The present Account is concerned with the use of the cluster approach in biocatalysis. The technical aspects of this methodology have been discussed in other reviews.^23−29^ For the purposes of this Account, a brief summary is sufficient.

**Figure 1:**
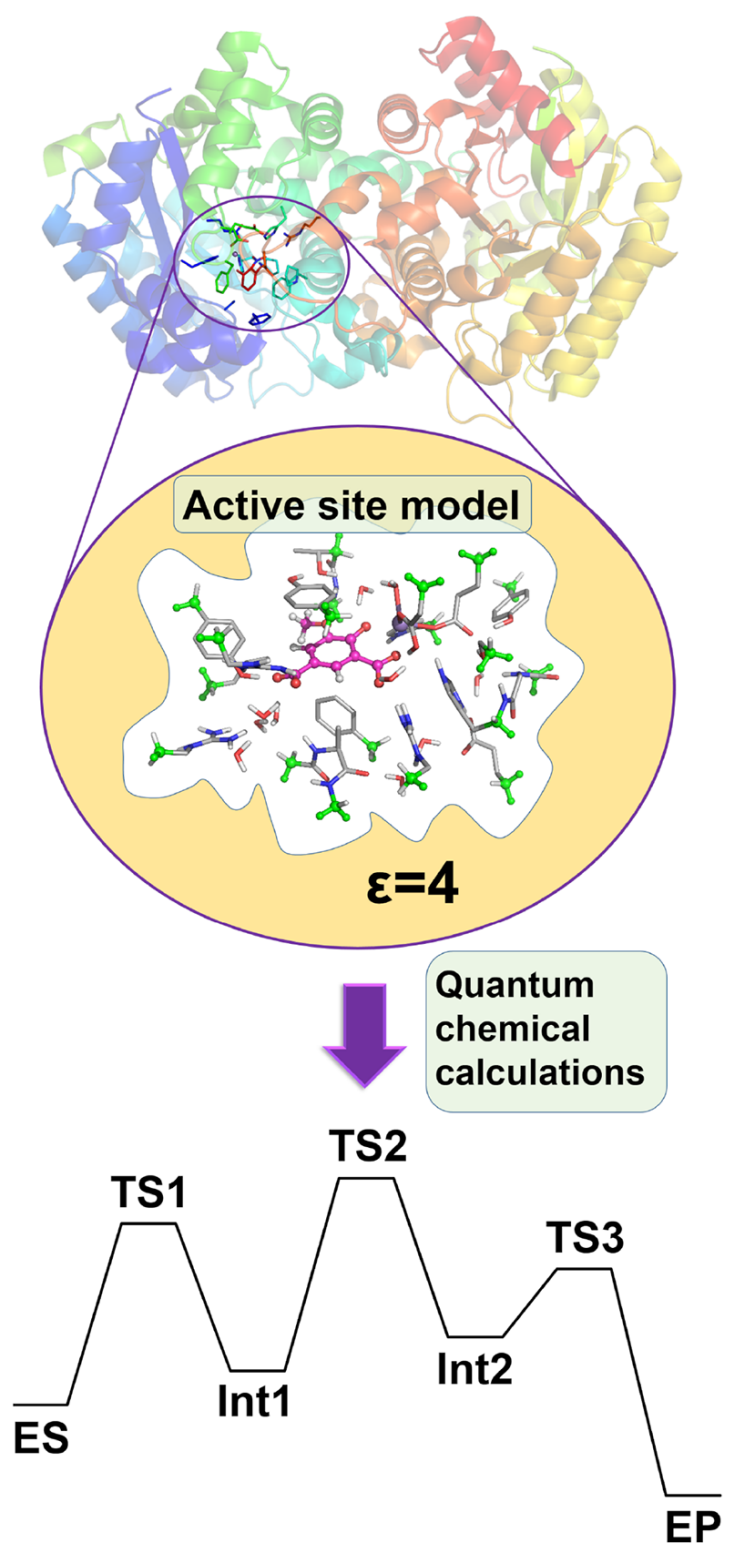
Schematic drawing of the cluster approach.

In the cluster approach, a relatively small part of the enzyme around the active site is selected as a model (Figure 1). Typically, a crystal structure is used to design the model, but other starting points can also be used, such as an MD simulation of the entire enzyme. Quantum chemical methods, most commonly density functional theory (DFT), are then used to calculate reaction energy profiles and other properties of the model. The parts of the enzyme that are left out are usually modeled using an implicit homogeneous solvation method, typically with a dielectric constant ε = 4. In addition, a number of centers at the edge of the model are kept fixed in the calculations in order to mimic the enzyme matrix around the active site and to prevent excessive movements of the various groups (Figure 1).

The cluster approach has proven to be a very effective method for mechanistic investigations.^23−29^ Even with rather small active site models, it has been possible to obtain valuable information about the reactions and to solve important mechanistic problems. Over the years, the models have gradually become larger and a great number of very diverse enzyme systems have been investigated.^23−29^

The models can today consist of more than 300 atoms and include typically the substrate(s), possible organic cofactors or metal ions with first-shell ligands, and residues directly involved in the reactions or interacting with the reacting species. The side chains are usually truncated to reduce the model size. Mechanistic investigations can beneficially start with a smaller preliminary model that allows for faster geometry optimization of intermediates and transition states (TSs) and thus faster screening of mechanistic alternatives. The information can then be transferred to the larger model, and comparison between models with different sizes can give insights into both the chemistry of the enzyme and the stability of the active site model.

The cluster approach deals typically with the chemical step of the enzyme reaction, starting with the substrate(s) already bound to the active site in the enzyme–substrate (ES) complex. Absolute binding free energies of substrates or products are not accessible using this methodology, and other computational approaches have to be employed if one is interested in these properties. Due to the limited size of the active site model, the specific effects of distant residues that are not included in the model, but that in some cases could be important for the catalytic activity, can of course not be reproduced. Similarly, allosteric effects cannot be modeled using the cluster approach. Other computational methods are more suitable for these purposes.

As mentioned above, DFT is the most common electronic structure method used in the cluster approach. Dispersion-corrected DFT has become the standard choice in recent years, in particular the hybrid functional B3LYP-D^30,31^ has been employed extensively in our work. This method is widely used in homogeneous catalysis modeling and provides a good balance between accuracy and speed. It has of course some inaccuracy, and one has in some cases to test other functionals or vary the amount of the exact exchange in B3LYP to assess the sensitivity of the results.^32^ Entropy effects are in general not included in the cluster approach, unless the specific problem requires it, such as in the case of the binding or release of gas molecules during the reaction. Likewise, tunneling effects are not considered.

The cluster approach has recently been employed in combination with other computational methodologies to obtain a more complete picture of enzyme reactions. For example, it has been combined with free energy perturbation (FEP) simulations to calculate relative binding affinities of substrates and products in the study of the mechanism of acyltransferase from *Mycobacterium smegmatis* (see below).^33^ It has also been used in conjunction with EVB simulations to investigate the role of entropy in enzyme catalysis.^34^

We have in recent years employed the cluster approach to study a number of enzymes of biocatalytic interest. These include: epoxide hydrolases,^35,36^ acyltransferases,^3,4,33,37^ decarboxylases,^1,38−43^ ω-transaminase,^44^ secondary alcohol dehydrogenase,^45^ imine reductase^46^ and Pictet–Spenglerases.^2,47^ One important recent development of relevance for biocatalysis is the ability to study enantioselectivity. The size of the models and the overall accuracy of the methods have been shown to be adequate to reproduce the stereochemical outcome and pinpoint its sources.^25^

In the following, selected examples from these studies will be discussed to highlight various aspects of the modeling methodology and to illustrate its capabilities and potential usefulness in biocatalytic applications. In all the examples discussed in this Account, the B3LYP functional^30^ was employed in conjunction with Grimme’s D3 dispersion correction^31^ (either already in the geometry optimization or as an a posteriori correction). Geometries were optimized with the 6-31G(d,p) for all atoms except metal ions, for which the lanl2dz was used. Energies were then evaluated using the larger basis set 6-311+G(2d,2p) for the nonmetal elements. Based on the optimized structures, solvation calculations using the SMD method^48^ (with ε = 4) were performed to estimate the surrounding effects of the protein environment. Zero-point energies were also included.

## 2 Substrate Binding

For small-molecule substrates, the cluster approach can provide valuable insights into the substrate binding mode(s), which can help to identify sites for the rational manipulation and redesign of the active site. Crystal structures of the enzymes with bound substrates or substrate analogues give of course important information in this regard. However, these are not always available, and, very importantly, such structures might not be representative for the catalytically productive binding mode of the substrate.

**Figure 2:**
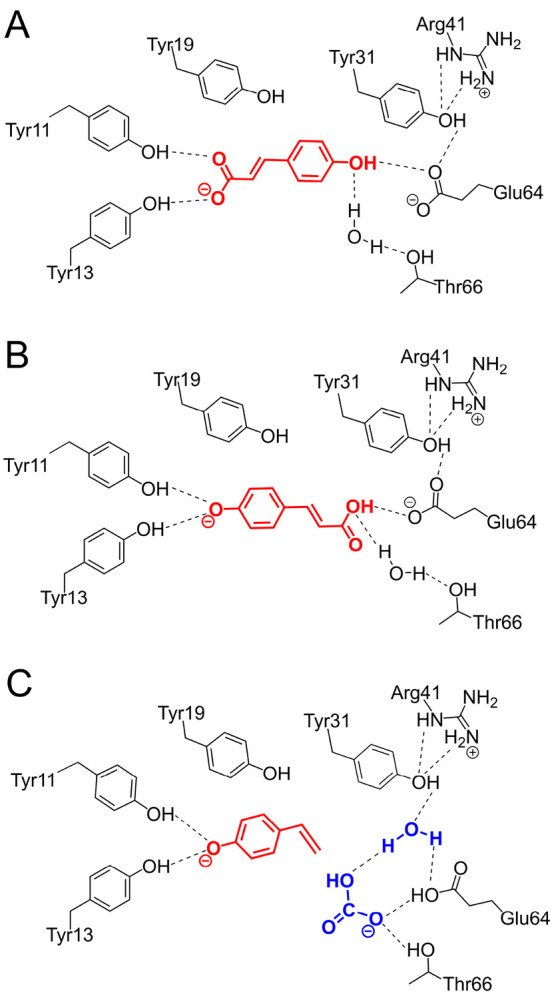
(A) Schematic drawing of the substrate binding mode of phenolic acid decarboxylase according to the crystal structure,^49^ (B) the binding mode obtained by the calculations,^38^ and (C) the binding mode of the three components in the hydration reaction.^1^

One illustrative example is phenolic acid decarboxylase (PAD), an enzyme that catalyzes the decarboxylation of phenolic acids to vinylphenols. Calculations using the cluster approach established that the binding mode of the natural substrate, *p*-coumaric acid, obtained in the crystal structure is not a productive one (Figure 2).^49^ Instead, another binding mode was identified in which the orientation of the substrate is flipped in the active site.^38^ Due to a better hydrogen-bonding network, the energy of this binding mode is almost 10 kcal/mol lower. Importantly, an energetically feasible reaction mechanism was obtained starting from the new binding mode, unlike the case of the previously proposed one.^38^

In general, the geometries of many enzyme–substrate complexes have to be optimized and their energies evaluated in order to identify the lowest-energy binding mode. The binding modes considered in this evaluation can differ in the orientation of the substrate in the active site pocket, in the hydrogen-bonding patterns between the substrate and active site residues and between the residues themselves, and also in the conformations and rotamers of the side chains of the active site amino acids. This procedure is of particular importance when studying the reactions of non-natural substrates, because the different size and shape compared to the natural substrates can lead to different binding modes. For example, in the study of the hydration activity of PAD, i.e., when the enzyme is used in the reverse direction to catalyze the hydration of hydroxystyrenes, more than 80 ES structures were considered because the non-natural substrates (*p*-vinylphenol, water, and a bicarbonate molecule) are smaller than the natural substrate and have therefore more flexibility in the active site pocket (Figure 2).^1^

**Figure 3:**
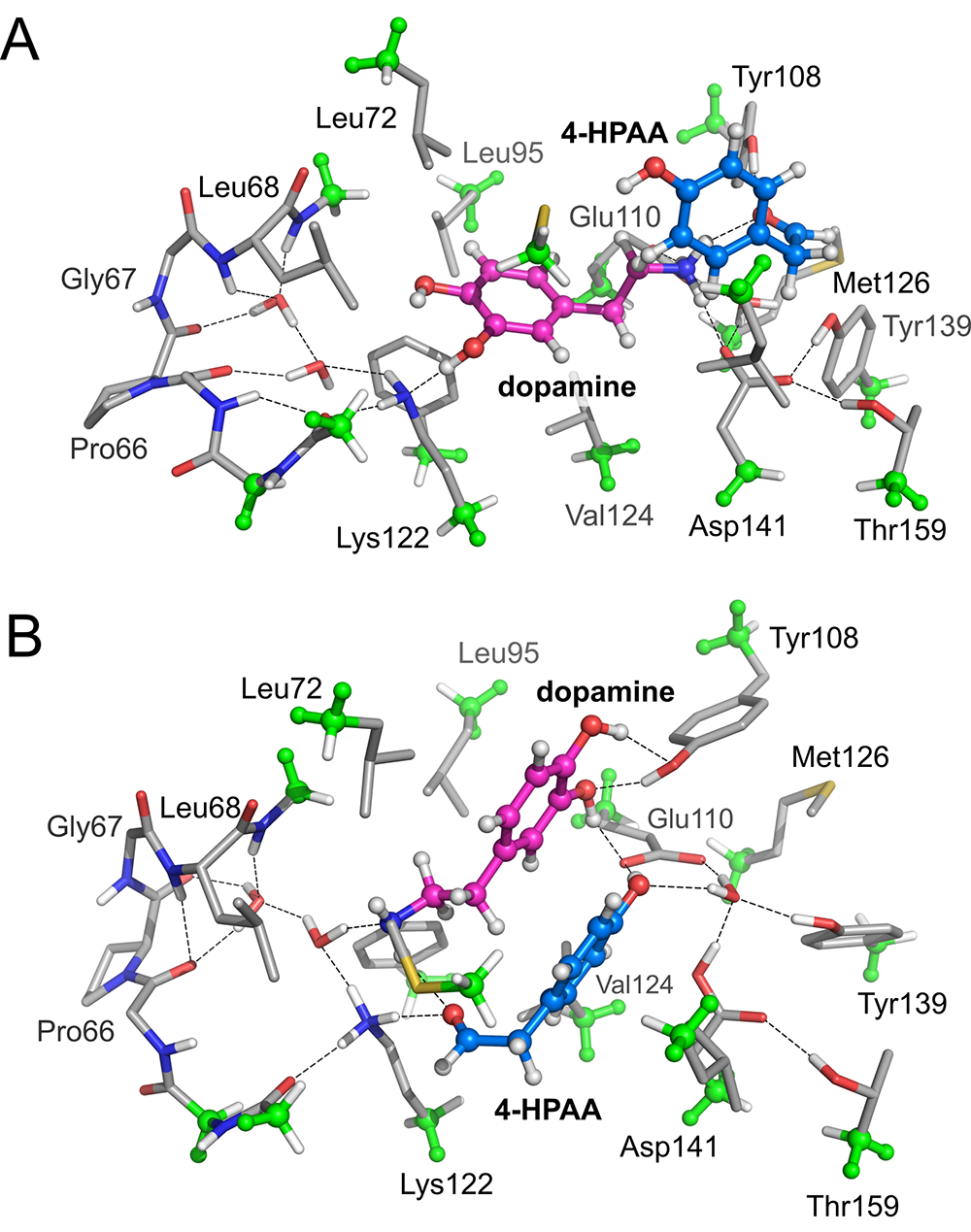
Optimized geometries of enzyme–substrate complexes of the two substrate binding modes of norcoclaurine synthase: (A) dopamine-first mode and (B) HPAA-first mode. The active site model consists of 304 atoms, and the centers fixed during the geometry optimizations are indicated in green.^2^

Another interesting case is norcoclaurine synthase, which catalyzes the condensation of dopamine with 4-hydroxyphenylacetaldehyde (4-HPAA) to yield (*S*)-norcoclaurine. Two substrate binding modes have been proposed on the basis of different crystal structures.^50^ These are called “dopamine-first” and “HPAA-first” binding modes and differ in the sequence of binding events, which has consequences for the reaction mechanism and the selectivity of the enzyme. Calculations with the cluster approach showed that the two binding modes (Figure 3) have essentially the same energy, differing by only 0.5 kcal/mol.^2^ However, the study of the complete reaction mechanism showed that only the dopamine-first mode is productive, with an energetically feasible overall barrier. The pathway starting from the HPAA-first binding mode had very high energies and could thus be ruled out.^2^

These examples show that knowledge about the substrate binding mode, either from experiments or calculations, should be complemented with a study of the full reaction in order to draw mechanistic conclusions. Typically, a number of low-energy ES complexes with different substrate binding modes have to be considered to ensure that the lowest-energy reaction pathway is obtained. This is especially important when studying selectivity, as it has been demonstrated that the substrate (or product) binding mode cannot always be used to predict the selectivity outcome of the enzyme reaction. One has to consider the entire reaction and deduce this information from the selectivity determining TS.^2,3,45−47^ For example, in a very recent study on the enantioselectivity of imine reductase from *Amycolatopsis orientalis*, the energy trend at the TS was found to be different compared to those of the ES and enzyme–product complexes.^46^

## 3 Reaction Mechanisms

Comprehensive knowledge about the reaction mechanisms constitutes a solid basis for the rational design of enzymes for biocatalytic applications. As discussed above, the cluster approach has proven very successful in elucidating reaction mechanisms of a wide range of enzymes.^23−29^ It can provide full energy profiles with detailed information about the reaction sequence, the natures of resting states and rate-determining steps, and geometries of all intermediates and TSs. The mechanistic information obtained from the calculations can be used to improve the biocatalytic applications of the enzymes. Phenolic acid decarboxylase can serve as an example here too.

**Figure 4:**
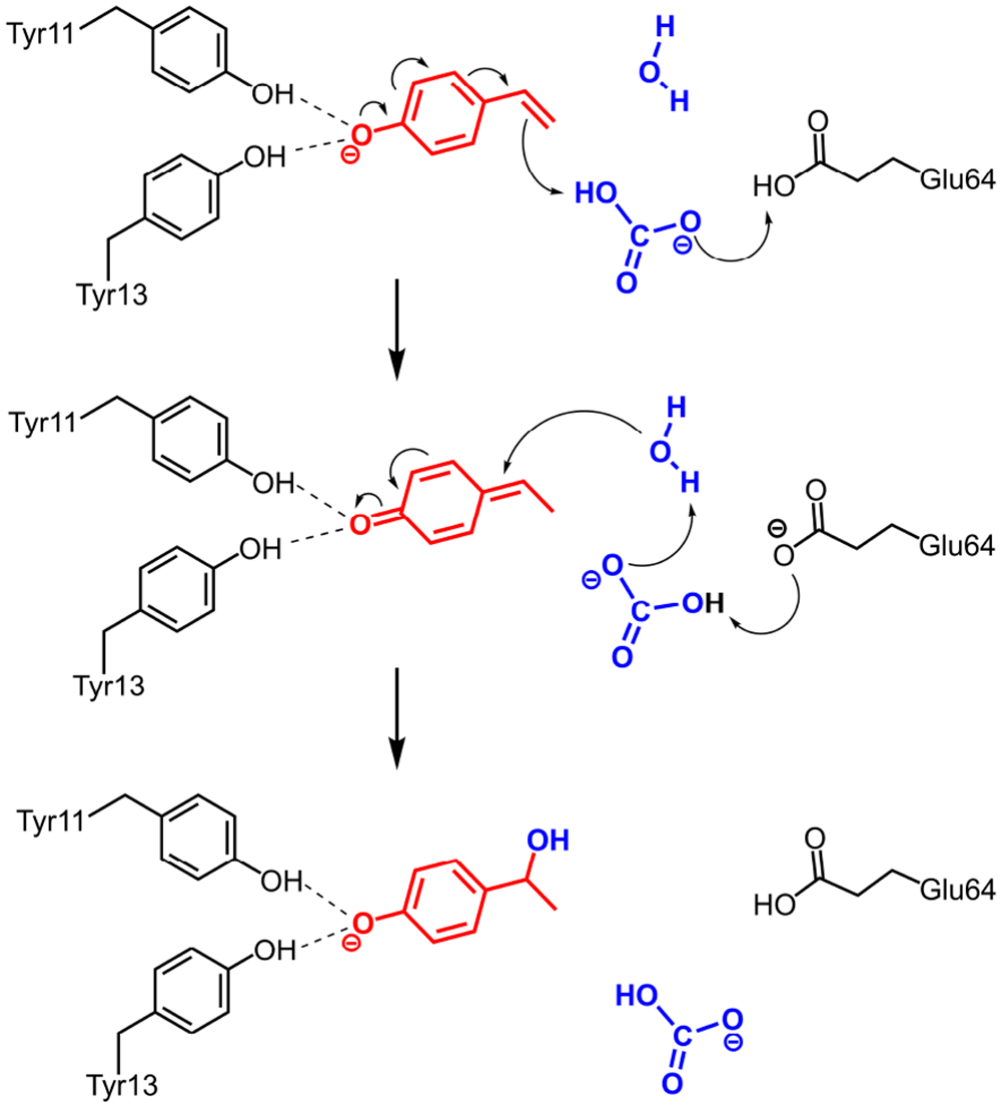
Reaction mechanism proposed on the basis of cluster calculations for the hydration reaction catalyzed by phenolic acid decarboxylase.^1^

As mentioned above, PADs have been shown to be capable of catalyzing the asymmetric hydration of styrene derivatives, which is an attractive protocol for synthesizing chiral alcohols by a direct hydration of C=C double bonds.^51^ This reaction was investigated with the cluster approach, and the mechanism proposed on the basis of these calculations is shown in Figure 4.^1^ As discussed above, a large number of ES complexes had to be investigated first, since the three substrates (*p*-vinylphenol, water and bicarbonate) can fit into the active site in many different ways. The reaction was then considered starting from the 20 lowest-energy ones.^1^

A previous proposal involving a C–C bond formation between bicarbonate and the styrene substrate could be ruled out on the basis of its high barrier. Instead, the calculations suggested that the bicarbonate functions as a proton shuttle between the Glu64 general acid and the styrene substrate (Figure 4). The water can then perform a nucleophilic attack on the resulting quinone methide intermediate to yield the alcohol product. The bicarbonate acts as a proton shuttle in the second step too, transferring a proton from the water to Glu64, which now functions as a general base to activate the nucleophile.^1^ This mechanism has a feasible energy barrier that is in good agreement with experimental data. Furthermore, the TS leading to the (*S*)-alcohol was calculated to be 2.3 kcal/mol lower than the TS leading to the (*R*)-alcohol, in good agreement with the 87% ee observed experimentally in favor of the former.^51^

The calculations showed that the same mechanism is valid also without the participation of the bicarbonate, as the barrier increased only by 0.7 kcal/mol when the bicarbonate was absent from the active site model. Interestingly, the calculated enantioselectivity-determining energy difference decreased to 1.2 kcal/mol without bicarbonate (experiments showed a racemic product). Detailed analysis of the TS geometries could provide a rationale for the observed selectivity.^1^

Consistently with the computational findings regarding the role of the bicarbonate in the catalysis and stereoselectivity, subsequent experimental work on ferulic acid decarboxylase showed that a strategically placed single-point mutation that introduces a carboxylate group into active site (Val46Glu or Val46Asp) leads to improved conversion and stereoselectivity.^52^ The carboxylate side chain can thus replace the bicarbonate as a proton shuttle and result in an efficient hydratase enzyme.

Another example of mechanistic work is metal-dependent decarboxylases from the amidohydrolase superfamily (AHS). These enzymes are of interest for biocatalytic applications because they can potentially catalyze the reverse reaction, i.e., the carboxylation of various substrates, providing a strategy to synthesize valuable chemicals by direct CO_2_ fixation.^53^

We have investigated the reaction mechanisms of a number of these enzymes using the cluster approach. They are 5-carboxyvanillate decarboxylase (LigW),^39^ γ-resorcylate decarboxylase (γ-RSD),^40^*iso*-orotate decarboxylase (IDCase),^41^ and 2,3-dihydroxybenzoic acid decarboxylase (2,3-DHBD).^42^ The calculations showed that they follow essentially the same mechanism (Table 1).^54^ The substrate binds to the divalent metal ion (Mn or Mg) in a bidentate manner, after which a proton transfer takes place from a metal-coordinated aspartic acid to the substrate. This is then followed by a C–C bond cleavage to form the corresponding product and release CO_2_. For LigW, γ-RSD, and 2,3-DHBD, the proton transfer was found to be rate-limiting, while for IDCase the C–C bond cleavage was rate-limiting.^54^

**Table 1:**
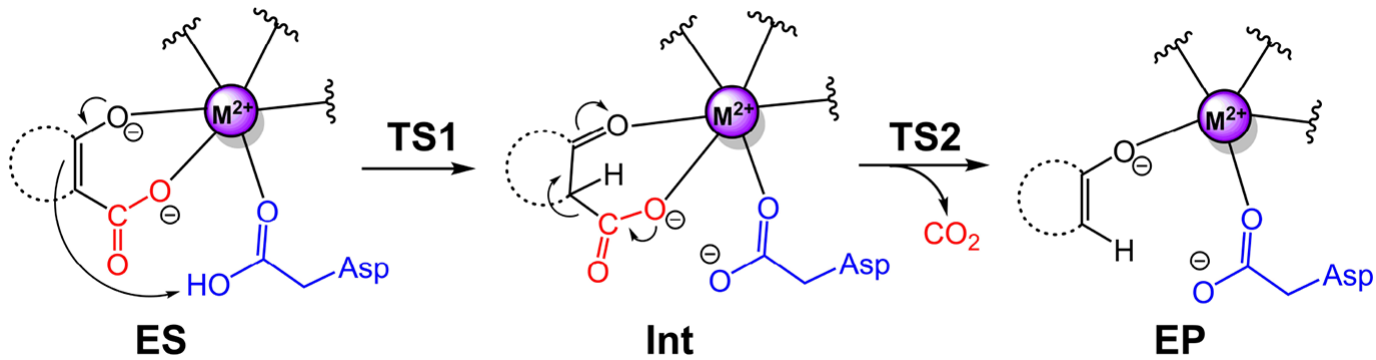

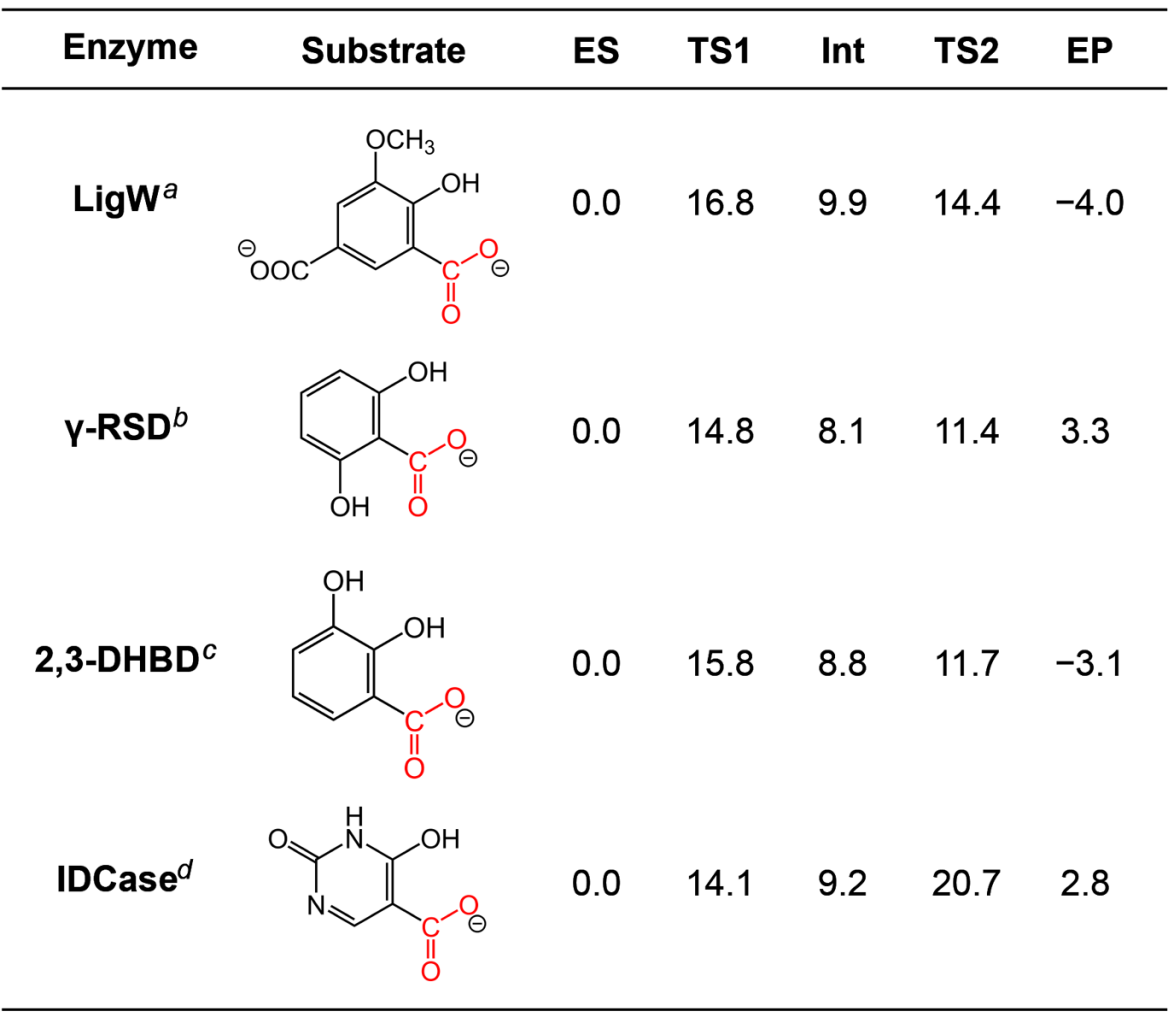
General Mechanism Suggested for Metal-Dependent Decarboxylases and Calculated Energies (kcal/mol) of the Intermediates and Transition States of Each Enzyme

a Ref (39).

b Ref (40).

c Ref (42).

d Ref (41).

An interesting experimental observation is that IDCase shows a narrower substrate scope compared to the other AHS decarboxylases, both for the decarboxylation reaction and the reverse carboxylation.^41^ Analysis of the substrate binding modes and the reaction energies helps to rationalize these observations. The calculations demonstrated that the natural substrate of IDCase, 5-carboxyuracil, binds to the metal in the productive bidentate binding mode, due to a number of specific interactions with surrounding active site residues. For the non-natural substrates, represented by γ-resorcylate, the nonproductive monodentate binding mode was calculated to be energetically much more stable, which results in a lack of reactivity.

Furthermore, the calculations showed that the activation barrier for IDCase is higher than for the other enzymes (Table 1), and the overall reaction is more exergonic. Combined, these two facts result in the reverse carboxylation reaction in IDCase being associated with a higher barrier than the other enzymes, explaining its lack of carboxylation activity.^41^

## 4 Enantioselectivity

As mentioned in the Introduction, the cluster models today are large enough to mimic the active site environment sufficiently accurately to be able to reproduce and even predict enantioselectivity for enzymes, at least for those with small-molecule substrates. Accordingly, the enantioselectivities of a number of enzymes of biocatalytic interest have been studied using this approach,^1,2,35−37,43,45−47^ and many of these examples have been reviewed recently.^25^ Here, the very recent work on strictosidine synthase (STR) will be discussed as an illustrative case.^47^

**Figure 5:**
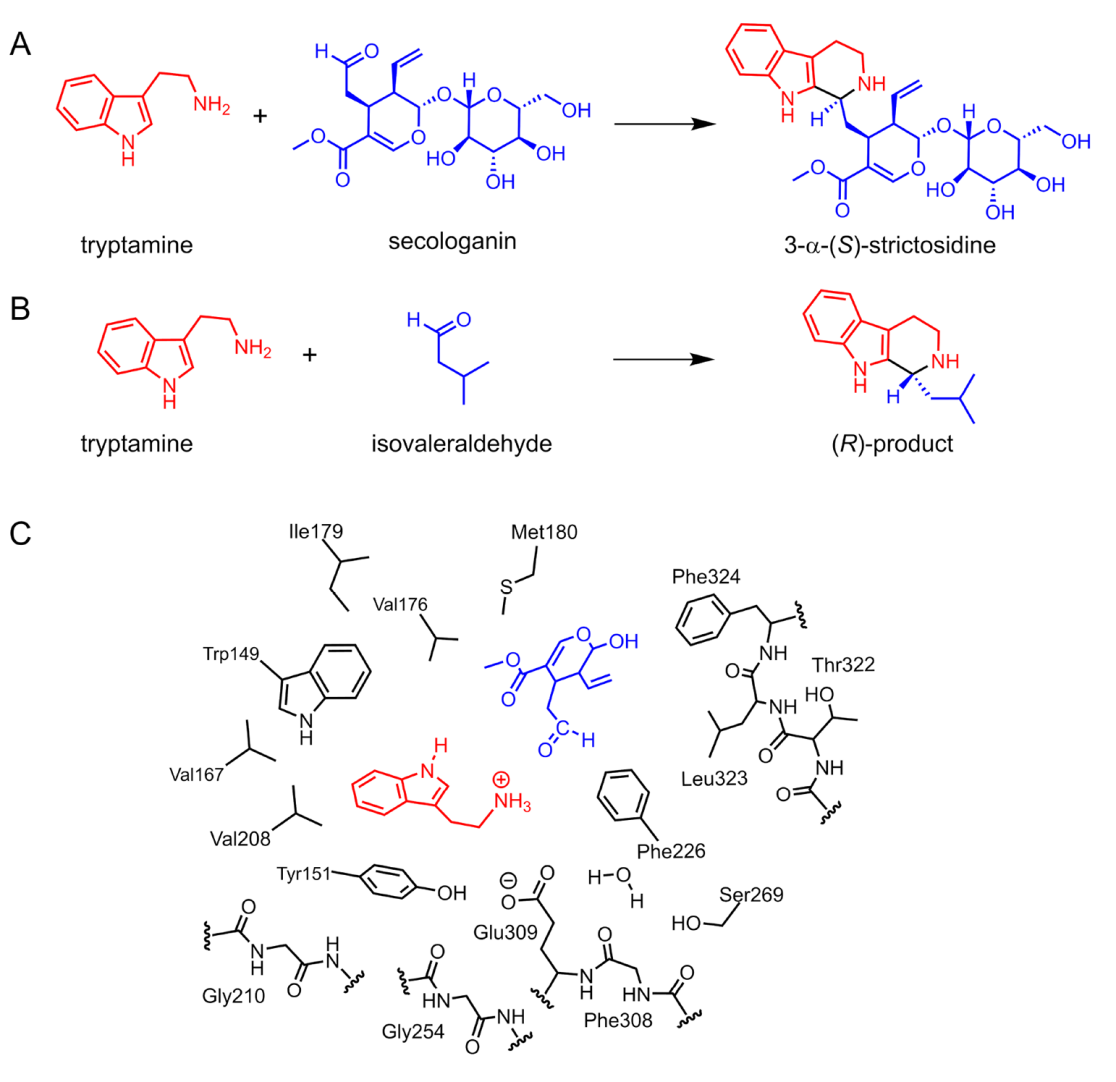
Strictosidine synthase-catalyzed reaction of tryptamine with (A) the natural substrate secologanin and (B) with the non-natural substrate isovaleraldehyde. (C) Schematic representation of the cluster model used in the calculations.^47^

STR catalyzes the Pictet–Spengler (PS) condensation between tryptamine and secologanin to yield (*S*)-strictosidine (Figure 5A). The enzyme is of interest in biocatalysis because it shows a relatively broad substrate scope, and synthetic strategies based on STR have been developed for the synthesis of various 1,2,3,4-tetrahydro-β-carbolines.^55^ An interesting feature of STR is that it displays different enantioselectivities for different substrates. For the natural substrates, tryptamine and secologanin, the reaction yields exclusively the (*S*)-enantiomer of the strictosidine product. The reactions of tryptamine with short-chain aliphatic aldehydes, on the other hand, favor the formation of the (*R*)-products (Figure 5B).^56^

We have investigated the detailed mechanism and the origins of enantioselectivity of the STR-catalyzed reaction, for both the natural and non-natural substrates, the latter represented by isovaleraldehyde which was found to yield the (*R*)-product with >98% ee (Figure 5B).^47^ Acetaldehyde, which yields a racemic product, was also considered in the calculations for comparison. A cluster model of the active site consisting of ca. 300 atoms was designed on the basis of the crystal structure, and the size of the secologanin substrate was reduced by truncating it at the glycosidic bond (Figure 5C).^47^

The calculations could first establish that STR follows the general mechanism of the PS reactions, showing high similarity to the norcoclaurine synthase reaction studied previously, in both the sequence of the elementary steps and the calculated energetics.^2^ The PS condensation starts by a proton transfer from the protonated amine group of the tryptamine substrate to a glutamate residue (Glu309), which is followed by hemiaminal formation between the two substrates. After a subsequent proton transfer process, the key iminium intermediate is formed by dehydration. A conformational change then takes place inside the active site to orientate the indole ring in a favorable position for the following cyclization. Finally, a proton transfer from the cyclized intermediate to Glu309 results in the formation of the strictosidine product.^47^

**Figure 6:**
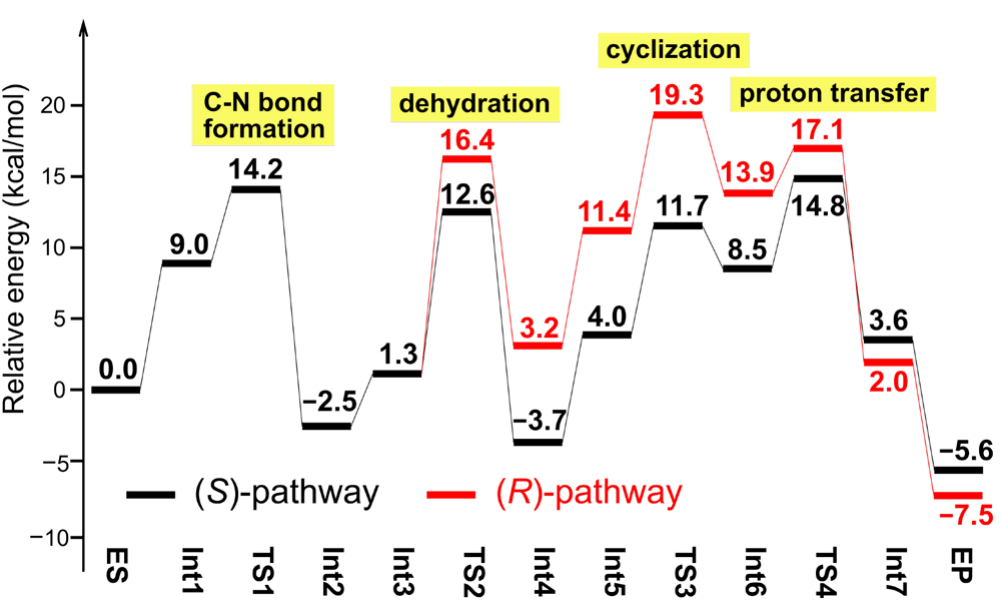
Calculated energy profiles for (*R*)- and (*S*)-pathways of the strictosidine synthase reaction for the natural substrates. Adapted with permission from ref (47). Copyright 2020 ACS Publications.

Very interestingly, the rate-determining step was found to be different in the path leading to the (*S*)-product compared to the one leading to the (*R*)-product (Figure 6). In the (*S*)-pathway the final proton transfer event (**TS4**) constitutes the highest barrier, while in the (*R*)-pathway the cyclization step (**TS3**) is the highest. The calculated energy difference between these two TSs (4.5 kcal/mol) is in agreement with the exclusive formation of the (*S*)-product observed experimentally.^57^

Importantly, the preference for the (*R*)-product for the non-natural substrate isovaleraldehyde could also be reproduced using the same active site model. The calculations showed a reversed preference by 2.4 kcal/mol, in good agreement with the experimental observations. Also for this substrate, the rate-determining step was found to be different for the two different pathways.^47^ In the case of acetaldehyde, the corresponding energy difference was calculated to be only 0.6 kcal/mol, which is consistent with the racemic outcome observed for this substrate.^56^

A detailed examination of the geometries of the key intermediates and TSs could identify the factors that contribute to these energy differences, and thus govern the selectivities for different substrates. These factors involve a combination of steric repulsions between the substrate and a number of the active site residues, the position of the large substituent in the forming six-membered ring in the cyclization step (being axial or equatorial), and the possibility to form an intramolecular hydrogen bond between different units of the substrates.^47^

This example demonstrates again the importance of considering the entire reaction in order to reproduce and rationalize the selectivity. It also highlights the value of studying several substrates, as the origins of the selectivity can change with different substrates.

## 5 Mutants

With the increasing model size, prediction of the effects of active site mutations on activity and selectivity using the cluster approach has become more accurate than before. A very recent example of the use of large cluster models to analyze mutational effects on enantioselectivity is the acyl transferase from *Mycobacterium smegmatis* (MsAcT). This enzyme is of interest in biocatalytic applications because of its ability to catalyze the transesterification reaction in both organic and aqueous media, with enantiopreference for a wide range of substrates.^58^

The cluster approach was used in combination with free energy perturbation simulations to investigate the details of the reaction mechanism.^33^ The calculations showed that the MsAcT reaction follows the general mechanism for enzymatic acyl transfer. It consists of two half-reactions: the acylation of the enzyme by an acyl-donor, and the acyl transfer from the acylated enzyme to the acyl-acceptor, with both half-reactions taking place via a tetrahedral intermediate. These calculations also addressed the issue of reaction specificity, i.e., the acyl transfer from the acylated enzyme taking place to either an alcohol acceptor or to water.^33^

A subsequent study focused on the enantioselectivity of MsAcT and considered the reactions of 1-isopropyl propargyl alcohol and 2-hydroxy propanenitrile as acyl acceptors.^3^ Although seemingly similar, these two substrates have opposite enantiopreferences, and the cluster calculations could reproduce this fact and provide a framework to understand the origins of the enantiopreference. These insights were later helpful in the rational design of variants of MsAcT with improved performance in terms of acyl transfer-to-hydrolysis ratio, substrate scope, and enantioselectivity.^59^

**Figure 7:**
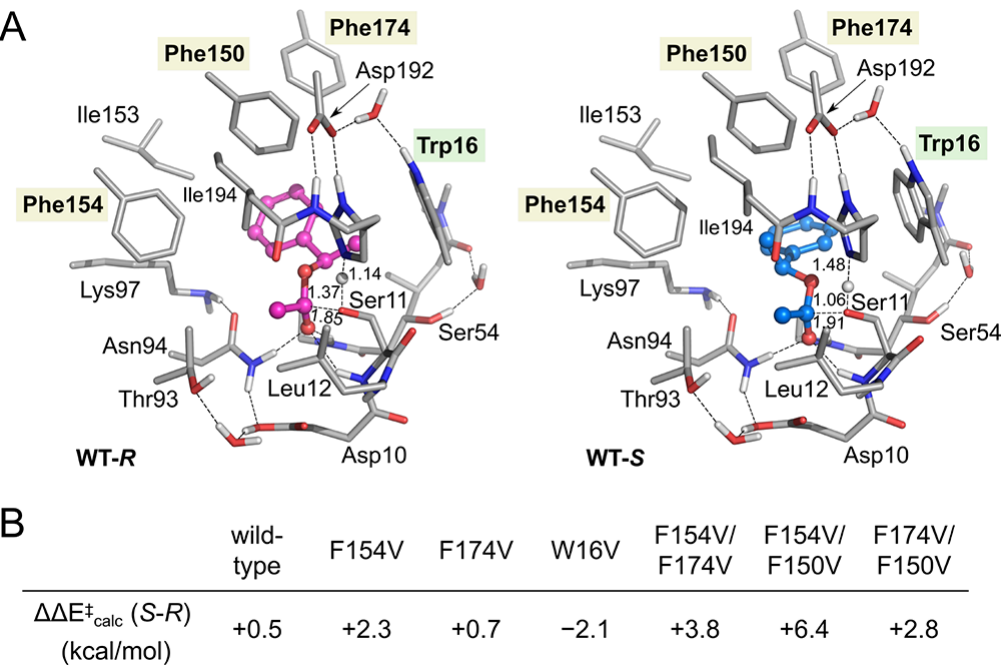
(A) Optimized structures of the selectivity-determining TSs for1-phenylethanol by the wild-type enzyme. (B) Effects of in silico mutations on the difference in selectivity-determining barrier.^37^

The results of the calculations were also used in the rational design of mutants of MsAcT with higher activity and enantioselectivity for bulky substrates.^37^ Further calculations were performed using 1-phenylethanol as a representative substrate, and the fact that the wild-type enzyme displays low activity and poor enantiopreference for this substrate could be reproduced (Figure 7).^60^ Namely, the barrier calculated for this substrate was found to be higher than the other studied substrates, and the difference between the barriers of the two enantiomers was found to be very small. Analysis of the substrate binding modes and the structures of the selectivity-determining TSs could rationalize these observations, and the insights were used to guide the design of a small library of single mutants that increased or decreased the steric demand in the different parts of the active site. Gratifyingly, a number of these experimentally examined mutants gave greatly increased conversions and enantioselectivities, for both 1-phenylethanol and other substrates.^37^ Further calculations on some of these mutants could explain the origins of their selectivities, and also predict the effects of some double mutants (Figure 7), which were then corroborated by experiments.^37^

The MsAcT case represents an example of how the cluster approach can be used to guide the rational design of enzyme variants to obtain improved properties. In general, the accurate prediction of mutational effects on activity and selectivity is not an easy task. Although the agreement with the experiments is not perfect, the trends are satisfactorily reproduced with the cluster models of today, which promises more applications of this kind in the future.

## 6 Conclusions

In this Account, we have discussed how the quantum chemical cluster approach can be utilized in the field of biocatalysis. Examples from our recent work have been selected to illustrate the capabilities and the status of the methodology in terms of providing knowledge about substrate binding, reaction mechanisms, origins of enantioselectivity and effects of mutations. Insights from the calculations into these features of the enzyme reactions are of great value for the rational design of variants with desired properties. We believe that the cluster approach will constitute a very useful tool in biocatalysis, complementing experiments and other computational techniques, and therefore, we expect that it will be used more in this field in the coming years.

## References

[ref1] Sheng X.; Himo F. Theoretical Study of Enzyme Promiscuity: Mechanisms of Hydration and Carboxylation Activities of Phenolic Acid Decarboxylase. ACS Catal. 2017, 7, 1733–1741. 10.1021/acscatal.6b03249.

[ref2] Sheng X.; Himo F. Enzymatic Pictet–Spengler Reaction: Computational Study of the Mechanism and Enantioselectivity of Norcoclaurine Synthase. J. Am. Chem. Soc. 2019, 141, 11230–11238. 10.1021/jacs.9b04591. 31265268

[ref3] Kazemi M.; Sheng X.; Himo F. Origins of Enantiopreference of *Mycobacterium Smegmatis* Acyl Transferase: A Computational Analysis. Chem.—Eur. J. 2019, 25, 11945–11954. 10.1002/chem.201902351. 31294500

[ref4] Sheng X.; Kazemi M.; Ża̧dło-Dobrowolska A.; Kroutil W.; Himo F. Mechanism of Biocatalytic Friedel–Crafts Acylation by Acyltransferase from *Pseudomonas Protegens*. ACS Catal. 2020, 10, 570–577. 10.1021/acscatal.9b04208. 31929947 PMC6945686

[ref5] Ferreira P.; Fernandes P. A.; Ramos M. J. Modern Computational Methods for Rational Enzyme Engineering. Chem. Catal. 2022, 2, 2481–2498. 10.1016/j.checat.2022.09.036.

[ref6] Derat E.; Kamerlin S. C. K. Computational Advances in Protein Engineering and Enzyme Design. J. Phys. Chem. B 2022, 126, 2449–2451. 10.1021/acs.jpcb.2c01198. 35387452

[ref7] Lovelock S. L.; Crawshaw R.; Basler S.; Levy C.; Baker D.; Hilvert D.; Green A. P. The Road to Fully Programmable Protein Catalysis. Nature 2022, 606, 49–58. 10.1038/s41586-022-04456-z. 35650353

[ref8] Klem H.; McCullagh M.; Paton R. S. Modeling Catalysis in Allosteric Enzymes: Capturing Conformational Consequences. Top. Catal. 2022, 65, 165–186. 10.1007/s11244-021-01521-1. 36304771 PMC9603403

[ref9] Wu L.; Qin L.; Nie Y.; Xu Y.; Zhao Y.-L. Computer-Aided Understanding and Engineering of Enzymatic Selectivity. Biotechnol. Adv. 2022, 54, 107793 10.1016/j.biotechadv.2021.107793. 34217814

[ref10] Planas-Iglesias J.; Marques S. M.; Pinto G. P.; Musil M.; Stourac J.; Damborsky J.; Bednar D. Computational Design of Enzymes for Biotechnological Applications. Biotechnol. Adv. 2021, 47, 107696 10.1016/j.biotechadv.2021.107696. 33513434

[ref11] Alonso-Cotchico L.; Rodríguez-Guerra J.; Lledós A.; Maréchal J.-D. Molecular Modeling for Artificial Metalloenzyme Design and Optimization. Acc. Chem. Res. 2020, 53, 896–905. 10.1021/acs.accounts.0c00031. 32233391

[ref12] Huggins D. J.; Biggin P. C.; Dämgen M. A.; Essex J. W.; Harris S. A.; Henchman R. H.; Khalid S.; Kuzmanic A.; Laughton C. A.; Michel J.; Mulholland A. J.; Rosta E.; Sansom M. S. P.; Van der Kamp M. W. Biomolecular Simulations: From Dynamics and Mechanisms to Computational Assays of Biological Activity. WIREs Comput. Mol. Sci. 2019, 9, e1393 10.1002/wcms.1393.

[ref13] Petrović D.; Kamerlin S. C. L. Molecular Modeling of Conformational Dynamics and its Role in Enzyme Evolution. Curr. Opin. Struct. Biol. 2018, 52, 50–57. 10.1016/j.sbi.2018.08.004. 30205262

[ref14] Ahmadi S.; Barrios Herrera L.; Chehelamirani M.; Hostaš J.; Jalife S.; Salahub D. R. Multiscale Modeling of Enzymes: QM-cluster, QM/MM, and QM/MM/MD: A Tutorial Review. Int. J. Quantum Chem. 2018, 118, e25558 10.1002/qua.25558.

[ref15] Wei W.-J.; Qian H.-X.; Wang W.-J.; Liao R.-Z. Computational Understanding of the Selectivities in Metalloenzymes. Front. Chem. 2018, 6, 638 10.3389/fchem.2018.00638. 30622942 PMC6308299

[ref16] Romero-Rivera A.; Garcia-Borràs M.; Osuna S. Computational Tools for the Evaluation of Laboratory-Engineered Biocatalysts. Chem. Commun. 2017, 53, 284–297. 10.1039/C6CC06055B. PMC5310519 27812570

[ref17] Sousa S. F.; Ribeiro A. J. M.; Neves R. P. P.; Brás N. F.; Cerqueira N. M. F. S. A.; Fernandes P. A.; Ramos M. J. Application of Quantum Mechanics/Molecular Mechanics Methods in the Study of Enzymatic Reaction Mechanisms. WIREs Comput. Mol. Sci. 2017, 7, e1281 10.1002/wcms.1281.

[ref18] Quesne M. G.; Borowski T.; de Visser S. P. Quantum Mechanics/Molecular Mechanics Modeling of Enzymatic Processes: Caveats and Breakthroughs. Chem.—Eur. J. 2016, 22, 2562–2581. 10.1002/chem.201503802. 26696271

[ref19] Wijma H. J.; Floor R. J.; Bjelic S.; Marrink S. J.; Baker D.; Janssen D. B. Enantioselective Enzymes by Computational Design and in Silico Screening. Angew. Chem., Int. Ed. Engl. 2015, 54, 3726–3730. 10.1002/anie.201411415. 25651000

[ref20] Brunk E.; Rothlisberger U. Mixed Quantum Mechanical/Molecular Mechanical Molecular Dynamics Simulations of Biological Systems in Ground and Electronically Excited States. Chem. Rev. 2015, 115, 6217–6263. 10.1021/cr500628b. 25880693

[ref21] Frushicheva M. P.; Mills M. J. L.; Schopf P.; Singh M. J.; Prasad R. B.; Warshel A. Computer Aided Enzyme Design and Catalytic Concepts. Curr. Opin. Chem. Biol. 2014, 21, 56–62. 10.1016/j.cbpa.2014.03.022. 24814389 PMC4149935

[ref22] Swiderek K.; Tuñón I.; Moliner V. Predicting Enzymatic Reactivity: from Theory to Design. WIREs Comput. Mol. Sci. 2014, 4, 407–421. 10.1002/wcms.1173.

[ref23] Himo F.; de Visser S. P. Status Report on the Quantum Chemical Cluster Approach for Modeling Enzyme Reactions. Commun. Chem. 2022, 5, 29 10.1038/s42004-022-00642-2. 36697758 PMC9814711

[ref24] Siegbahn P. E. M. A Quantum Chemical Approach for the Mechanisms of Redox-Active Metalloenzymes. RSC Adv. 2021, 11, 3495–3508. 10.1039/D0RA10412D. 35424322 PMC8694229

[ref25] Sheng X.; Kazemi M.; Planas F.; Himo F. Modeling Enzymatic Enantioselectivity using Quantum Chemical Methodology. ACS Catal. 2020, 10, 6430–6449. 10.1021/acscatal.0c00983.

[ref26] Himo F. Recent Trends in Quantum Chemical Modeling of Enzymatic Reactions. J. Am. Chem. Soc. 2017, 139, 6780–6786. 10.1021/jacs.7b02671. 28493715

[ref27] Blomberg M. R. A.; Borowski T.; Himo F.; Liao R.-Z.; Siegbahn P. E. M. Quantum Chemical Studies of Mechanisms for Metalloenzymes. Chem. Rev. 2014, 114, 3601–3658. 10.1021/cr400388t. 24410477

[ref28] Siegbahn P. E. M.; Himo F. The Quantum Chemical Cluster Approach for Modeling Enzyme Reactions. Wiley Interdiscip. Rev.: Comput. Mol. Sci. 2011, 1, 323–336. 10.1002/wcms.13.

[ref29] Himo F. Quantum Chemical Modeling of Enzyme Active Sites and Reaction Mechanisms. Theor. Chem. Acc. 2006, 116, 232–240. 10.1007/s00214-005-0012-1.

[ref30] aBecke A. D. Density functional Thermochemistry. III. The Role of Exact Exchange. J. Chem. Phys. 1993, 98, 5648–5652. 10.1063/1.464913.

[ref31] aGrimme S.; Antony J.; Ehrlich S.; Krieg H. A Consistent and Accurate Ab Initio Parametrization of Density Functional Dispersion Correction (DFT-D) for the 94 Elements H–Pu. J. Chem. Phys. 2010, 132, 154104 10.1063/1.3382344. 20423165

[ref32] Siegbahn P. E. M.; Blomberg M. R. A. A Systematic DFT Approach for Studying Mechanisms of Redox Active Enzymes. Front. Chem. 2018, 6, 644 10.3389/fchem.2018.00644. 30627530 PMC6309562

[ref33] Kazemi M.; Sheng X.; Kroutil W.; Himo F. Computational Study of *Mycobacterium Smegmatis* Acyl Transferase Reaction Mechanism and Specificity. ACS Catal. 2018, 8, 10698–10706. 10.1021/acscatal.8b03360.

[ref34] Kazemi M.; Himo F.; Åqvist J. Enzyme Catalysis by Entropy without Circe Effect. Proc. Natl. Acad. Sci. U.S.A. 2016, 113, 2406–2411. 10.1073/pnas.1521020113. 26755610 PMC4780625

[ref35] Lind M. E. S.; Himo F. Quantum Chemistry as a Tool in Asymmetric Biocatalysis: Limonene Epoxide Hydrolase Test Case. Angew. Chem., Int. Ed. 2013, 52, 4563–4567. 10.1002/anie.201300594. PMC3734700 23512539

[ref36] Lind M. E. S.; Himo F. Quantum Chemical Modeling of Enantioconvergency in Soluble Epoxide Hydrolase. ACS Catal. 2016, 6, 8145–8155. 10.1021/acscatal.6b01562.

[ref37] Jost E.; Kazemi M.; Mrkonjić V.; Himo F.; Winkler C. K.; Kroutil W. Variants of the Acyltransferase from *Mycobacterium smegmatis* Enable Enantioselective Acyl Transfer in Water. ACS Catal. 2020, 10, 10500–10507. 10.1021/acscatal.0c02981.

[ref38] Sheng X.; Lind M. E. S.; Himo F. Theoretical Study of the Reaction Mechanism of Phenolic Acid Decarboxylase. FEBS J. 2015, 282, 4703–4713. 10.1111/febs.13525. 26408050

[ref39] Sheng X.; Zhu W.; Huddleston J.; Xiang D. F.; Raushel F. M.; Richards N. G.; Himo F. A Combined Experimental-Theoretical Study of the LigW-Catalyzed Decarboxylation of 5-Carboxyvanillate in the Metabolic Pathway for Lignin Degradation. ACS Catal. 2017, 7, 4968–4974. 10.1021/acscatal.7b01166.

[ref40] Sheng X.; Patskovsky Y.; Vladimirova A.; Bonanno J. B.; Almo S. C.; Himo F.; Raushel F. M. Mechanism and Structure of γ-Resorcylate Decarboxylase. Biochemistry 2018, 57, 3167–3175. 10.1021/acs.biochem.7b01213. 29283551 PMC5988983

[ref41] Sheng X.; Plasch K.; Payer S. E.; Ertl C.; Hofer G.; Keller W.; Braeuer S.; Goessler W.; Glueck S. M.; Himo F.; Faber K. Reaction Mechanism and Substrate Specificity of *Iso*-orotate Decarboxylase: A Combined Theoretical and Experimental Study. Front. Chem. 2018, 6, 608 10.3389/fchem.2018.00608. 30619817 PMC6305744

[ref42] Hofer G.; Sheng X.; Braeuer S.; Payer S. E.; Plasch K.; Goessler W.; Faber K.; Keller W.; Himo F.; Glueck S. M. Metal Ion Promiscuity and Structure of 2,3-Dihydroxybenzoic Acid Decarboxylase of *Aspergillus oryzae*. ChemBioChem. 2021, 22, 652–656. 10.1002/cbic.202000600. 33090643 PMC7894528

[ref43] aPlanas F.; Sheng X.; McLeish M. J.; Himo F. A Theoretical Study of the Benzoylformate Decarboxylase Reaction Mechanism. Front. Chem. 2018, 6, 205 10.3389/fchem.2018.00205. 29998094 PMC6028569

[ref44] aCassimjee K. E.; Manta B.; Himo F. A Quantum Chemical Study of the ω-Transaminase Reaction Mechanism. Org. Biomol. Chem. 2015, 13, 8453–8464. 10.1039/C5OB00690B. 26154047

[ref45] Moa S.; Himo F. Quantum Chemical Study of Mechanism and Stereoselectivity of Secondary Alcohol Dehydrogenase. J. Inorg. Biochem. 2017, 175, 259–266. 10.1016/j.jinorgbio.2017.07.022. 28803132

[ref46] Prejanò M.; Sheng X.; Himo F. Computational Study of Mechanism and Enantioselectivity of Imine Reductase from *Amycolatopsis orientalis*. ChemistryOpen 2022, 11, e202100250 10.1002/open.202100250. 34825518 PMC8734122

[ref47] Sheng X.; Himo F. Computational Study of Pictet–Spenglerase Strictosidine Synthase: Reaction Mechanism and Origins of Enantioselectivity of Natural and Non-Natural Substrates. ACS Catal. 2020, 10, 13630–13640. 10.1021/acscatal.0c03758.

[ref48] Marenich A. V.; Cramer C. J.; Truhlar D. G. Universal Solvation Model Based on Solute Electron Density and on a Continuum Model of the Solvent Defined by the Bulk Dielectric Constant and Atomic Surface Tensions. J. Phys. Chem. B 2009, 113, 6378–6396. 10.1021/jp810292n. 19366259

[ref49] Frank A.; Eborall W.; Hyde R.; Hart S.; Turkenburg J. P.; Grogan G. Mutational Analysis of Phenolic Acid Decarboxylase from *Bacillus subtilis* (*Bs*PAD), Which Converts Bio-derived Phenolic Acids to Styrene Derivatives. Catal. Sci. Technol. 2012, 2, 1568–1574. 10.1039/c2cy20015e.

[ref50] aIlari A.; Franceschini S.; Bonamore A.; Arenghi F.; Botta B.; Macone A.; Pasquo A.; Bellucci L.; Boffi A. Structural Basis of Enzymatic (*S*)-Norcoclaurine Biosynthesis. J. Biol. Chem. 2009, 284, 897–904. 10.1074/jbc.M803738200. 19004827

[ref51] Wuensch C.; Gross J.; Steinkellner G.; Gruber K.; Glueck S. M.; Faber K. Asymmetric Enzymatic Hydration of Hydroxystyrene Derivatives. Angew. Chem., Int. Ed. 2013, 52, 2293–2297. 10.1002/anie.201207916. 23335002

[ref52] Payer S. E.; Pollak H.; Glueck S. M.; Faber K. A Rational Active-Site Redesign Converts a Decarboxylase into a C=C Hydratase: “Tethered Acetate” Supports Enantioselective Hydration of 4-Hydroxystyrenes. ACS Catal. 2018, 8, 2438–2442. 10.1021/acscatal.7b04293. 29527405 PMC5838639

[ref53] aPayer S. E.; Faber K.; Glueck S. M. Non–Oxidative Enzymatic (De) Carboxylation of (Hetero) Aromatics and Acrylic Acid Derivatives. Adv. Synth. Catal. 2019, 361, 2402–2420. 10.1002/adsc.201900275. 31379472 PMC6644310

[ref54] Sheng X.; Himo F. Mechanisms of Metal-dependent Non-redox Decarboxylases from Quantum Chemical Calculations. Comput. Struct. Biotechnol. J. 2021, 19, 3176–3186. 10.1016/j.csbj.2021.05.044. 34141138 PMC8187880

[ref55] aCigan E.; Eggbauer B.; Schrittwieser J. H.; Kroutil W. The Role of Biocatalysis in the Asymmetric Synthesis of Alkaloids – An Update. RSC Adv. 2021, 11, 28223–28270. 10.1039/D1RA04181A. 35480754 PMC9038100

[ref56] Pressnitz D.; Fischereder E.-M.; Pletz J.; Kofler C.; Hammerer L.; Hiebler K.; Lechner H.; Richter N.; Eger E.; Kroutil W. Asymmetric Synthesis of (*R*)-1-Alkyl-Substituted Tetrahydro-β-carbolines Catalyzed by Strictosidine Synthases. Angew. Chem., Int. Ed. 2018, 57, 10683–10687. 10.1002/anie.201803372. PMC6146909 29852524

[ref57] Stöckigt J.; Zenk M. H. Strictosidine (isovincoside): The Key Intermediate in the Biosynthesis of Monoterpenoid Indole Alkaloids. J. Chem. Soc., Chem. Commun. 1977, 646–648. 10.1039/C39770000646.

[ref58] Cannazza P.; Donzella S.; Pellis A.; Contente M. L. *Mycobacterium smegmatis* Acyltransferase: The Big New Player in Biocatalysis. Biotechnol. Adv. 2022, 59, 107985 10.1016/j.biotechadv.2022.107985. 35609801

[ref59] Godehard S. P.; Badenhorst C. P. S.; Müller H.; Bornscheuer U. T. Protein Engineering for Enhanced Acyltransferase Activity, Substrate Scope, and Selectivity of the *Mycobacterium Smegmatis* Acyltransferase MsAcT. ACS Catal. 2020, 10, 7552–7562. 10.1021/acscatal.0c01767.

[ref60] de Leeuw N.; Torrelo G.; Bisterfeld C.; Resch V.; Mestrom L.; Straulino E.; van der Weel L.; Hanefeld U. Ester Synthesis in Water: *Mycobacterium Smegmatis* Acyl Transferase for Kinetic Resolutions. Adv. Synth. Catal. 2018, 360, 242–249. 10.1002/adsc.201701282.

